# Effects of a Nonwearable Digital Therapeutic Intervention on Preschoolers With Autism Spectrum Disorder in China: Open-Label Randomized Controlled Trial

**DOI:** 10.2196/45836

**Published:** 2023-08-24

**Authors:** Liting Chu, Li Shen, Chenhuan Ma, Jinjin Chen, Yuan Tian, Chuncao Zhang, Zilan Gong, Mengfan Li, Chengjie Wang, Lizhu Pan, Peiying Zhu, Danmai Wu, Yu Wang, Guangjun Yu

**Affiliations:** 1 Department of Child Health Care, Shanghai Children’s Hospital School of Medicine Shanghai Jiao Tong University Shanghai China; 2 Division of Child and Adolescent Health Shanghai Municipal Center for Disease Control and Prevention Shanghai China; 3 Clinical Research Center Shanghai Jiao Tong University Affiliated Sixth People’s Hospital Shanghai China; 4 School of Medicine The Chinese University of Hong Kong Shenzhen China

**Keywords:** autism spectrum disorder, digital therapy, nonwearable, preschoolers, randomized controlled trial, autism, neurodevelopmental disorder, difficulty with communication, social interaction, ADHD, attention-deficit/hyperactivity disorder, digital therapy, digital intervention

## Abstract

**Background:**

Autism spectrum disorder (ASD) is a neurodevelopmental disorder that can cause difficulty with communication and social interactions as well as complicated family dynamics. Digital health interventions can reduce treatment costs and promote healthy lifestyle changes. These therapies can be adjunctive or replace traditional treatments. However, issues with cooperation and compliance prevent preschool patients with ASD from applying these tools. In this open-label, randomized controlled trial, we developed a nonwearable digital therapy called virtual reality–incorporated cognitive behavioral therapy (VR-CBT).

**Objective:**

The aim of this study was to assess the adjunctive function of VR-CBT by comparing the effects of VR-CBT plus learning style profile (LSP) intervention with those of LSP-only intervention in preschool children with ASD.

**Methods:**

This trial was performed in China on 78 preschool children (age 3-6 years, IQ>70) diagnosed with ASD who were randomized to receive a 20-week VR-CBT plus LSP intervention (intervention group, 39/78, 50%) or LSP intervention only (control group, 39/78, 50%). The primary outcome was the change of scores from baseline to week 20, assessed by using the parent-rated Autism Behavior Checklist (ABC). Secondary outcomes included the Childhood Autism Rating Scale (CARS), Attention-Deficit/Hyperactivity Disorder Rating Scale-IV (ADHD-RS-IV), and behavioral performance data (accuracy and reaction time) in go/no-go tasks. All primary and secondary outcomes were analyzed in the intention-to-treat population.

**Results:**

After the intervention, there was an intervention effect on total ABC (β=–5.528; *P*<.001) and CARS scores (β=–1.365; *P*=.02). A similar trend was observed in the ABC subscales: sensory (β=–1.133; *P*=.047), relating (β=–1.512; *P*=.03), body and object use (β=–1.211; *P*=.03), and social and self-help (β=–1.593; *P*=.03). The intervention also showed statistically significant effects in improving behavioral performance (go/no-go task, accuracy, β=2.923; *P*=.04). Moreover, a significant improvement of ADHD hyperactivity-impulsivity symptoms was observed in 53 children with comorbid ADHD based on ADHD-RS-IV (β=–1.269; *P*=.02). No statistically significant intervention effect was detected in the language subscale of ABC (β=–.080; *P*=.83). Intervention group girls had larger improvements in ABC subscales, that is, sensory and body and object use and in the CARS score and accuracy of go/no-go task (all *P*<.05) than the control group girls. Statistically significant intervention effects could be observed in hyperactivity-impulsivity symptoms in the intervention group boys with comorbid ADHD compared with those in the control group boys (β=–1.333; *P*=.03).

**Conclusions:**

We found potentially positive effects of nonwearable digital therapy plus LSP on core symptoms associated with ASD, leading to a modest improvement in the function of sensory, motor, and response inhibition, while reducing impulsivity and hyperactivity in preschoolers with both ASD and ADHD. VR-CBT was found to be an effective and feasible adjunctive digital tool.

**Trial Registration:**

Chinese Clinical Trial Registry ChiCTR2100053165; http://www.chictr.org.cn/showproj.aspx?proj=137016

## Introduction

Autism spectrum disorder (ASD) is a type of neurodevelopmental disorder that is typically diagnosed during childhood, characterized by social deficits, narrowed interests, as well as repetitive and stereotyped behaviors [1-3]. The incidence of ASD is increasing year by year, and the 2020 National Multicenter Population Study stated that the incidence rate of ASD in Chinese children aged 6 to 12 years was 0.70% [4]. The overall incidence of ASD in China ranks first in the world, thereby becoming an important public health problem, as this disorder can place a heavy burden on families and society [4-6]. A study on the Chinese family life quality of preschool children with ASD reported that the severity of the social deficits exhibited by patients with ASD was correlated with a decrease in the quality of family life and increase in parenting burdens [7].

Early interventions can reduce the degree of obstacles in adapting and mastering behavioral skills in children with ASD [8]. Considering that drug treatments cannot fundamentally improve the symptoms of social deficits, narrowed interests, or repetitive stereotyped behaviors [9], nonpharmacological options such as behavioral and educational options are treated as first-line therapies [10]. Digital therapies or digital health interventions (DHIs) are software-based therapeutic options that provide patients with alternative interventions focused on preventing and managing diseases. User-centered approaches to DHI designs can make it easier for people to engage with interventions that have the potential to improve their health [11]. The rapid development of digital health in recent years has ushered in novel opportunities for innovation in the field of ASD rehabilitation. The effectiveness of digital therapies as interventions for ASD has been supported by randomized controlled trials and meta-analyses [12].

Virtual reality (VR) technology is a form of digital therapy using computer technology to create realistic visual, audio, tactile, and other integrated virtual environments. With immersive, interactive, and highly perceptive characteristics, VR has become a robust DHI tool [13], offering a safe, repeatable, and diversifiable environment platform for those with ASD to learn during treatment [14,15]. For instance, McCleery et al [16] assessed a VR-based police safety interaction training program in populations with ASD. Their results confirmed the feasibility, safety, and utility of that intervention for verbally fluent adolescents and adults with ASD. Vahabzadeh and colleagues [17] designed the Empowered Brain System, a combination of modern smart glasses and educational modules targeted at teaching socioemotional and behavioral management skills. A game named Face2Face was also utilized to encourage face-directed gaze during social interactions. In that study, after the use of smart glasses was implemented, progress in attention and social function was reported. These existing works have tentatively confirmed the effects of VR as an intervention tool for improving socialization, understanding, and perception skills in patients with ASD.

Early interventions are critical for young populations with ASD [8]. However, most VR-related studies have focused on school-age participants or on those who had already reached adulthood [12], highlighting that more studies are required to understand the effectiveness of the application of VR tools in preschool children. The current interventions are not suitable for preschool children with ASD for the following reasons. First, the existing VR tools require children to wear the technology, and oversized VR tools can cause fatigue and even dizziness in children, which negatively affects the immersive experience of the virtual world [18]. Moreover, wearable devices such as head-mounted displays are not recommended for children younger than 7 years, as their visual centers are still developing [19,20]. Second, a single VR scene can easily lead children with ASD to develop fears of difficulty, escape emotions, and even aggravate stereotyped behaviors, thereby weakening the effect of the intervention [21]. These obstacles make it very difficult to apply VR tools in preschoolers with ASD due to issues with cooperation and compliance.

To overcome these disadvantages of wearable VR tools, we designed a nonwearable VR intervention platform (VR-incorporated cognitive behavioral therapy [VR-CBT]) with interventions of both sensory and motor stimulation for preschool participants with ASD specifically. This tool incorporates multiple games, which treat cognitively impaired areas via selectively activating specific cognitive systems in the brain. Considering the development manifestation of autism, we introduced learning style profile (LSP) training as the other intervention. LSP is a condensed version of the SCERTS (Social Communication Emotional Regulation Transactional Support) training model extracted by Dr Patrick J Rydell of the Rocky Mountain Autism Center in the United States for the multidimensional comprehensive correction of children with autism [22]. LSP combines the fundamental difficulties of autism with the standards for diagnostic and treatment. Every child has a unique learning style that can be thought of as methods or preferences for acquiring knowledge from their environment and fostering social relationships. However, the ability of children with ASD to pay attention to others in their environment may be severely restricted due to variations in learning styles, which may further results in impairments of their engagement and ability to learn social interactions. These youngsters may struggle to establish common meanings, shared feelings, shared affect, and eventually, customary behaviors as a result of learning style issues. Children with ASD have unconventional learning patterns, making social interaction difficult. Instead of trying to teach children with ASD all they need to know, the LSP intervention tries to illustrate different patterns, tactics, and preferences to show how they learn and acquire knowledge from their surroundings [23]. The effectiveness of LSP has been demonstrated in our previous research [24,25]. In this study, we aimed to assess the adjunctive function of VR-CBT on preschool children with ASD by comparing the effect of VR-CBT plus LSP intervention versus LSP intervention alone.

## Methods

### Ethics Approval

This open-label randomized controlled trial was organized in Shanghai Children’s Hospital, China. Recruitment of the study participants began in November 2021, and data collection was completed in October 2022. This study was approved by the ethics committee of Shanghai Children's Hospital (approval 2021R064) and registered at the Chinese Clinical Trial Registry (ChiCTR2100053165) on November 13, 2021.

### Participant Recruitment and Eligibility

Participating children were recruited through notices or advertisements in outpatient clinics, kindergartens, and communities. Parents could contact the research staff if they were interested in this project as well as provide informed consent. Children who met the criteria on Autism Diagnostic Observation Schedule, 2nd edition [26] and had a diagnosis of ASD from the pediatrician based on the Diagnostic and Statistical Manual of Mental Disorders, 5th edition [27] as well as met the following inclusion criteria were enrolled in this study: (1) age 3-6 years, (2) IQ>70 assessed by the Wechsler Preschool and Primary Scale of Intelligence (Chinese revision) [28], (3) ability to understand instructions with the assistance of the research staff, and (4) did not participate in any training course recently. Children were excluded if they had other neurological or psychiatric diseases (eg, childhood schizophrenia, obsessive compulsive disorder, unclassified generalized developmental disorders, Rett syndrome, child disintegrating mental disorders, mental retardation).

### Randomization and Masking

Participants who met all the eligibility criteria and provided written informed consent were randomly assigned (1:1) to receive VR-CBT plus LSP [22] (intervention) or LSP only (control) by using a computer-generated randomization sequence. The allocation sequence was stratified for co-occurring attention-deficit/hyperactivity disorder (ADHD). Randomization was performed by research staff using SAS statistics software (version 9.4; SAS Institute Inc). Research staff received sequentially numbered, opaque, sealed envelopes containing treatment tasks. After children with ASD enrolled in this study, the research staff opened a sequentially numbered envelope containing a card that determined whether the patient entered the intervention group or the control group. Participants, research staff, and pediatricians were aware of group allocation. Analyses were performed by a statistician masked to group allocation.

### Interventions

#### Design

Three therapists with primary health qualification certificates or above and majors in children’s speech-language and cognitive behavior training with more than 3 years of working experience were part of this project. Each child was accompanied by the same therapist to the intervention session, and each intervention contained 1 child and 1 therapist. Children in the intervention group completed 40 sessions (2 sessions per week; 20 minutes of VR-CBT and 1 hour of LSP per session) in 20 weeks. Those who were in the control group were only required to complete the LSP course in 40 sessions (2 sessions per week; 1 hour of LSP per session) in 20 weeks.

#### VR-CBT Intervention

**Figure 1:**
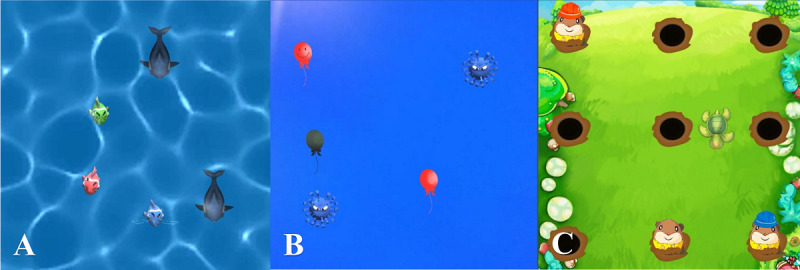
Three game scenes (computer-displayed) in virtual reality–incorporated cognitive behavioral therapy. Participants need to follow the correct instructions to earn scores. (A) Underwater World: The participant has to step on the red fish without glasses in order to score points. (B) Sky Balloons: The participant is instructed to step on the red balloon with no expression. (C) Rural Gophers: The participant is instructed to step on gophers without glasses or hats. Scores will be deducted if the participant makes the wrong decision.

**Figure 2:**
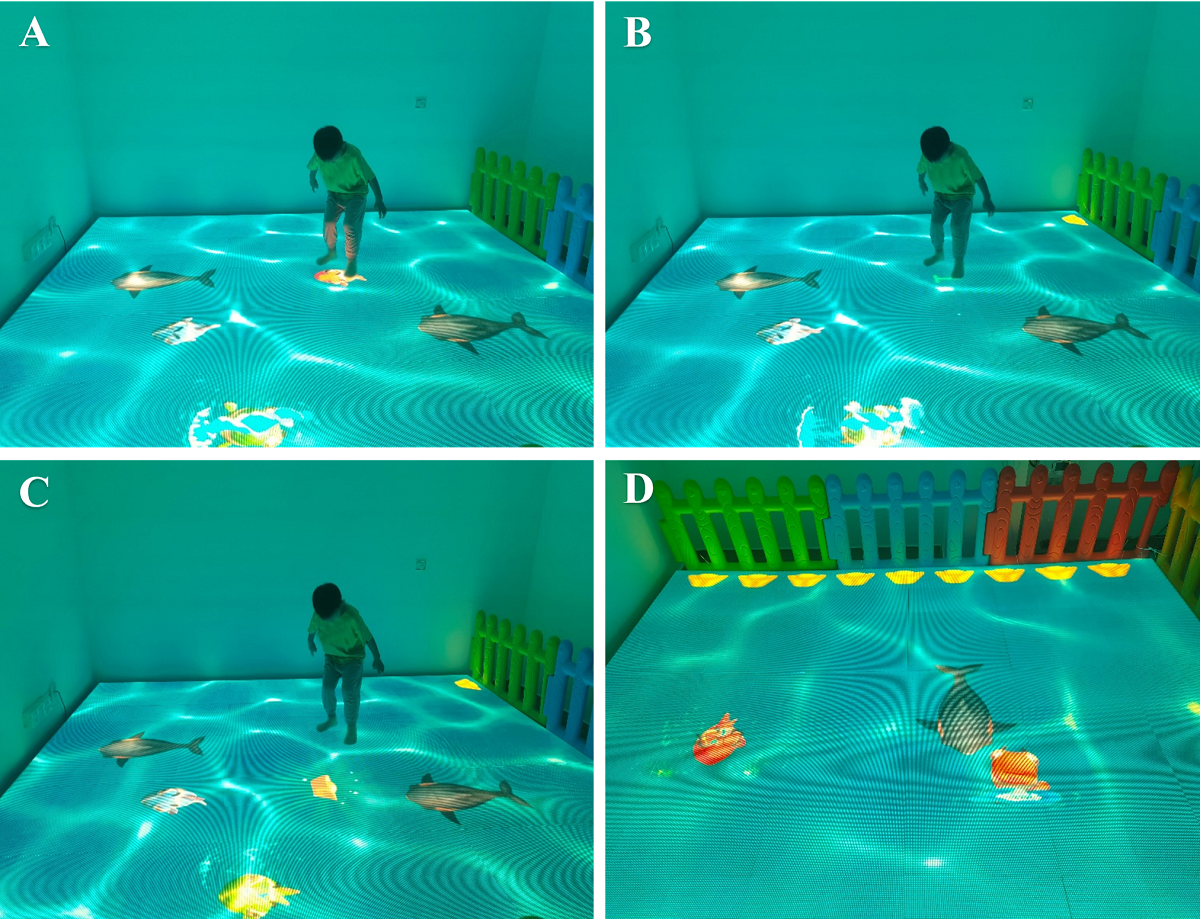
Process of the Underwater World game. (A) The participant has to run toward a red fish and step on it after observing it carefully and waiting patiently. (B) Correct actions are rewarded with scores shown in green, but if the participant does not choose correctly, points are deducted in red from the score. (C) When the participant achieves 5 points, he or she receives a gold bar (upper right). (D) When the participant plays the game according to the instructions, the top corner is filled with gold bars.

VR-CBT is a research-based digital treatment that combines immersive interactive video game technology with applied behavior analysis and sensory integration training. By precisely constructing sensitive motor nerve stimulation methods, this intervention could selectively engage the brain’s cognitive nervous system and increase children’s ability to process and respond to information. Interventions were conducted using a desktop computer (Lenovo) and a light-emitting diode floor tile screen (3 m × 3 m) with a resolution of P3.91, surrounded by guardrails and walls. The content displayed by the computer was projected onto the floor tile screen. We established a database for those who received the VR-CBT intervention. The therapist entered the participant identifier and frequency at each session. VR-CBT consists of 3 scenes (Figure 1 and Figure 2) and 6 steps: game perception, rule communication, interactive communication, cognitive training, reaction inhibition, and comprehensive feedback. The steps, photos, contents, specific actions, and examples of the games in this intervention are presented in Multimedia Appendix 1.

#### LSP Intervention

LSP consisted of top 10 learning style components. Each component contained a large arrow divided into 4 quadrants: I don’t know, I know a bit, I am starting to use, and I can use habitually. These quadrants represented the continuous development of the learning style process of children with ASD. Each capability in the LSP component changed from less to more so that the children could achieve the balance of capabilities [22]. Before training, the therapist identified the representative learning style characteristics of the participants together with parents and set realistic rehabilitation goals. Parents could be involved in the entire intervention and could choose to carry out training for children at home by themselves.

### Outcomes and Measures

The children’s basic information was collected from their parents at baseline. The questionnaire requested information on children’s age, gender, father and mother’s education, parental time with children, etc. Parents also had to complete scale assessments. Children finished the go/no-go task with the guidance of the research staff before the intervention. Posttesting occurred no more than 2 weeks following the last intervention session. Questionnaires of baseline and outcome measures were collected online. All scales were completed with detailed explanation from the same research staff (Multimedia Appendix 2).

The primary outcome measure was the mean change in the Autism Behavior Checklist (ABC) [29] scores from those before the intervention to those after the intervention. The ABC is a 57-item behavior rating scale with 5 subscales, namely, sensory (9 items), relating (12 items), body and object use (12 items), language (13 items), and social and self-help (11 items). The total score (range from 0 to 158) is obtained by adding the weight of different subscales. The higher scale scores correlate with more severe symptoms.

The secondary outcomes measured in this study consisted of changes in the Childhood Autism Rating Scale (CARS) [30], ADHD Rating Scale-IV (ADHD-RS-IV; total, inattentive, and hyperactive/impulsive scales) for those with co-occurring ADHD [31], and behavioral performance data (accuracy and response time) in the go/no-go task [32]. The reliability and validity of the ABC, CARS, and ADHD-RS-IV in the Chinese version have shown good reliability and validity [33,34].

### Sample Size Calculation and Statistical Analysis

G*Power software (version 3.1.9; Heinrich Heine University) was used to estimate the required sample size. We estimated that the effect size of the changes in the primary outcome ABC total was 0.30 based on α=.05 and β=.80 in repeated-measures ANOVA. Thus, 34 children were required for each group. Considering a sample loss rate of 10%, an additional 4 participants were required for each group. Therefore, the total sample for this study was 76, with 38 participants in each group.

Shapiro-Wilk test was used to test the normality of the data. The normally distributed variables were expressed as mean (SD) and categorical variables as frequency (%). Two-sided Student *t* tests and chi-square tests were used to compare the differences between group differences. Since the generalized estimating equations (GEEs) can utilize the information from incomplete pairs of observations despite the missing value [35], we employed GEE to evaluate the interactions between group and time as well as changes of measurements between group differences. The distributional family was Gaussian, with an identity link and exchangeable correlation matrix. The intention-to-treat principle was applied. Analyses were performed using SPSS software (version 26; IBM Corp) and R software (version 4.2.1; R Foundation for Statistical Computing). A *P* value of less than .05 was considered statistically significant.

## Results

### Participant Characteristics

**Figure 3:**
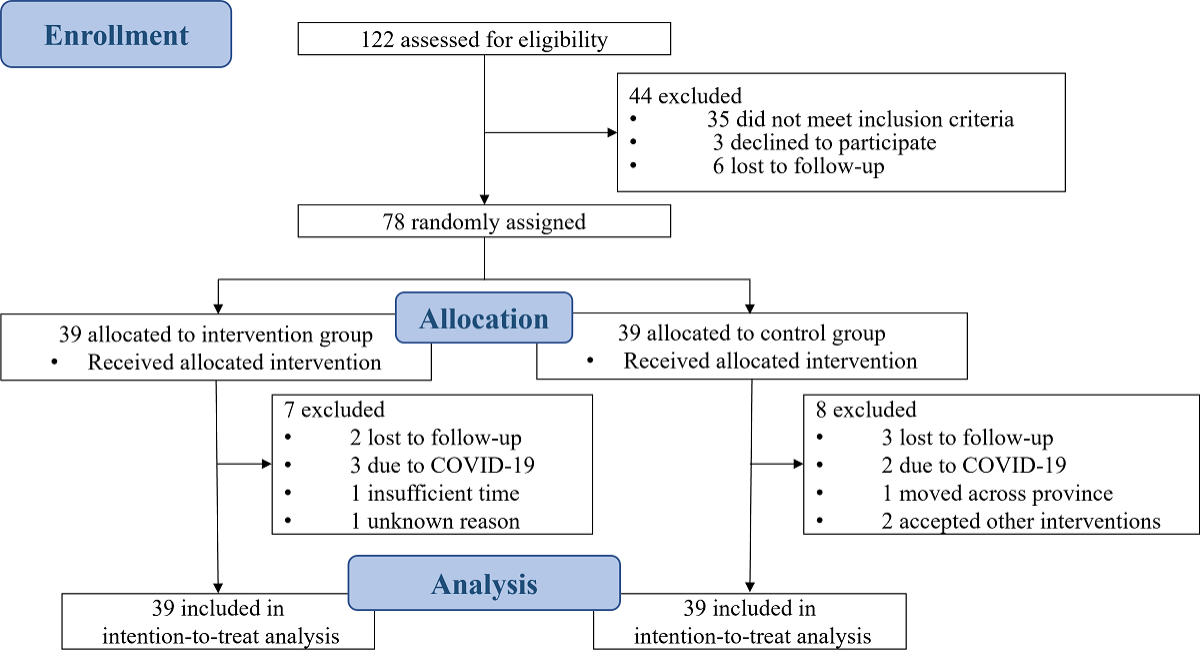
CONSORT (Consolidated Standards for Reporting Trials) flowchart of participants selected to participate in this study.

A total of 122 children were assessed for eligibility, but 44 (35 did not meet inclusion criteria, 3 declined to participate, and 6 lost to follow-up) were excluded at baseline assessment. Seventy-eight children who met the inclusion criteria were randomly assigned to either the intervention group (39/78, 50%) or the control group (39/78, 50%). The CONSORT (Consolidated Standards for Reporting Trials) flowchart in Figure 3 provides the details of the participants (Multimedia Appendix 3). The baseline characteristics of the participants were well-balanced between the 2 groups. The mean age of the 78 participants was 5.02 (SD 0.82) years; 78% (61/78) were boys and 68% (53/78) had combined ADHD. The age of children with co-occurring ADHD ranged from 4.16 to 6.00 years, with mean age of 5.02 (SD 0.52) years. In the intervention group, 69% (27/39) of the children had ADHD, while in the control group, 67% (26/39) of the children had ADHD. There were no statistically significant differences in ABC, CARS, and ADHD-RS-IV scores between the 2 groups at baseline (all *P>*.05, see Table 1).

**Table 1:** Baseline characteristics of the participants.

			Intervention group (n=39)		Control group (n=39)		Total (N=78)
Age (years), mean (SD)			5.00 (0.82)		5.03 (0.83)		5.02 (0.82)
Boys, n (%)			31 (80)		30 (77)		61 (78)
Girls, n (%)			8 (21)		9 (23)		17 (22)
**Father’s education, n (%)**							
	Graduate or above	3 (8)		10 (26)		13 (17)	
	Undergraduate	35 (90)		28 (72)		63 (81)	
	High school or below	1 (3)		1 (3)		2 (3)	
**Mother’s education, n (%)**							
	Graduate or above	5 (13)		2 (5)		7 (9)	
	Undergraduate	31 (80)		27 (69)		58 (74)	
	High school or below	3 (8)		10 (26)		13 (17)	
**Parental time with children, n (%)**							
	≥3 h/d	28 (72)		34 (87)		62 (82)	
	<3 h/d	11 (28)		5 (13)		16 (21)	
**Children’s exposure to electronic screen time, n (%)**							
	>1 h/d	16 (41)		13 (33)		29 (37)	
	0.5-1 h/d	16 (41)		17 (44)		33 (42)	
	<0.5 h/d	7 (18)		9 (23)		16 (21)	
**Parent-child communication time, n (%)**							
	1-2 d/w	1 (3)		2 (5)		3 (4)	
	3-4 d/w	4 (10)		0 (0)		4 (5)	
	All day	34 (87)		37 (95)		71 (91)	
Attention-deficit/hyperactivity disorder comorbidity, n (%)			27 (69)		26 (67)		53 (68)
**Autism Behavior Checklist** **scores, mean (SD)**							
	Total	76.26 (14.85)		73.74 (11.15)		75.00 (13.11)	
	Sensory	13.85 (4.13)		12.85 (2.96)		13.35 (3.61)	
	Relating	19.41 (4.64)		18.23 (4.65)		18.82 (4.65)	
	Body and object use	12.31 (3.42)		12.62 (3.17)		12.46 (3.28)	
	Language	16.62 (4.62)		15.95 (3.91)		16.28 (4.26)	
	Social and self-help	14.08 (4.52)		14.10 (3.57)		14.09 (4.05)	
Childhood Autism Rating Scale scores, mean (SD)			36.69 (4.11)		36.90 (4.31)		36.79 (4.19)
**Attention-Deficit/Hyperactivity Disorder Rating Scale-IV** **scores, mean (SD)**							
	Total	36.59 (2.76)		37.58 (2.94)		37.08 (2.87)	
	Inattention	18.74 (1.79)		19.69 (1.93)		19.21 (1.91)	
	Hyperactivity-impulsivity	17.85 (1.77)		17.88 (1.61)		17.87 (1.68)	

Of the 78 randomized children, 63 (81%) completed the intervention and postassessments (intervention, n=32; control, n=31). Attrition included 7 intervention group children (2 were lost to follow-up, 3 due to COVID-19, 1 did not have sufficient time, and the other withdrew for unknown reasons) and 8 control group children (3 were lost to follow-up, 2 due to COVID-19, 1 moved across province, and 2 accepted other interventions).

### Evaluation of the Intervention

#### Intervention Effects on Symptoms by ABC and CARS Analyses

**Figure 4:**
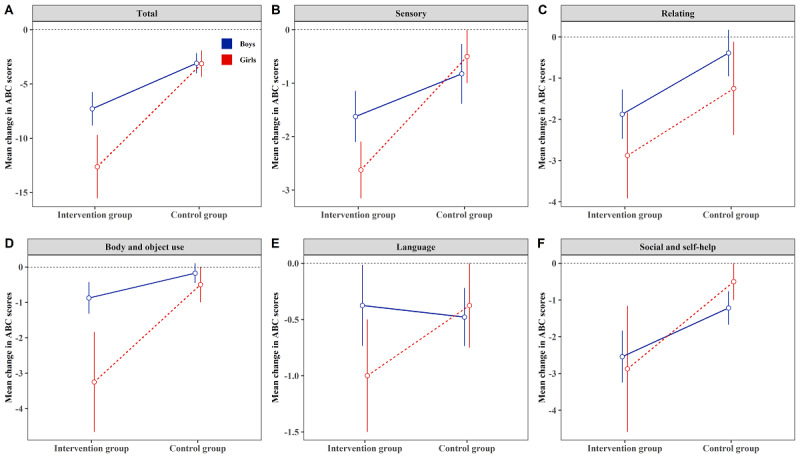
Mean (SE) change in the Autism Behavior Checklist scores from those before the intervention to those after the intervention in each group in the intention-to-treat population stratified by sex. ABC: Autism Behavior Checklist.

The results of ABC and CARS scores are presented in Table 2. Significant group × time difference was found in total ABC (β=–5.746; *P<*.001) and ABC subscales, namely, sensory, relating, body and object use, and social and self-help (β=–1.244, –1.513, –1.189, and –1.457, respectively; all *P<*.05). Significant interaction effect was also found in CARS (β=–1.304; *P=*.03). Compared with the control group, the intervention group showed a significant improvement in total ABC (β*=*–5.528; *P<*.001); ABC subscales, that is, sensory (β*=*–1.133; *P=*.047), relating (β*=*–1.512; *P=*.03), body and object use (β*=*–1.211; *P=*.03), and social and self-help (β*=*–1.593; *P=*.03); and CARS (β*=*–1.365; *P=*.02) after the intervention, whereas no significant intervention effect was observed for language in the ABC scale (group × time: β=–.172; *P=*.63; changes: β=–.080; *P=*.83). Multimedia Appendix 4 clearly describes the changes before and after the intervention in total ABC scores and subscales of both groups. When the sample was stratified by sex, we only noted interaction effect and group difference in total ABC (β=–4.612 and –4.205 respectively; all *P<*.05) among boys. Additionally, in line with the interaction plots (Figure 4), both intervention and control groups did not show gender difference in changes of ABC subscales (all *P*>.05), although some subscales showed a small trend for higher score changes in girls. However, girls in the intervention group had generally higher score changes; therefore, the magnitude of the effect became significant in the ABC subscales of sensory (β*=*–2.125; *P=*.002), body and object use (β*=*–2.750; *P=*.05), and CARS (β=–2.375; *P=*.03) (Table 2).

**Table 2:** Effects of the intervention on Autism Behavior Checklist scores, Childhood Autism Rating Scale scores, and measurements in go/no-go tasks, analyzed by generalized estimating equations.

Outcome measures					Group by time interaction effect							Change in baseline intervention group versus control group			
					β^a^ (95% CI)			*P* value			β (95% CI)				*P* value
**Autism Behavior Checklist**															
	Total			–5.746 (–8.681 to –2.812)			<.001			–5.528 (–8.599 to –2.457)				<.001	
	Sensory			–1.244 (–2.329 to –.158)			.03			–1.133 (–2.251 to –.016)				.047	
	Relating			–1.513 (–2.854 to –.173)			.03			–1.512 (–2.904 to –.120)				.03	
	Body and object use			–1.189 (–2.252 to –.125)			.03			–1.211 (–2.295 to –.126)				.03	
	Language			–.172 (–.866 to .521)			.63			–.080 (–.784 to .625)				.83	
	Social and self-help			–1.457 (–2.876 to –.038)			.04			–1.593 (–3.056 to –.130)				.03	
Childhood Autism Rating Scale					–1.304 (–2.462 to –.146)			.03			–1.365 (–2.534 to –.196)				.02
**Go/no-go task**															
	Accuracy (%)			2.258 (–.520 to 5.036)			.11			2.923 (.090 to 5.757)				.04	
	Reaction time (s)			–3.105 (–9.197 to 2.986)			.32			–3.171 (–9.271 to 2.929)				.31	
**Boys**															
	**Autism Behavior Checklist**														
		Total	–4.612 (–7.909 to –1.316)			.01			–4.205 (–7.656 to –.754)				.02		
		Sensory	–1.027 (–2.386 to .332)			.14			–.799 (–2.211 to .613)				.27		
		Relating	–1.432 (–2.929 to .065)			.06			–1.484 (–3.060 to .092)				.07		
		Body and object use	–.704 (–1.700 to .293)			.17			–.701 (–1.713 to .310)				.17		
		Language	–.016 (–.850 to .818)			.97			.103 (–.747 to .953)				.81		
		Social and self-help	–1.243 (–2.807 to .321)			.12			–1.324 (–2.935 to .287)				.11		
	Childhood Autism Rating Scale			–.950 (–2.304 to .403)			.17			–1.022 (–2.392 to .349)				.14	
	**Go/no-go task**														
		Accuracy (%)	.681 (–2.547 to 3.908)			.68			1.548 (–1.773 to 4.869)				.36		
		Reaction time (s)	–2.301 (–9.107 to 4.505)			.51			–2.427 (–9.274 to 4.419)				.49		
**Girls**															
	**Autism Behavior Checklist**														
		Total	–9.386 (–15.143 to –3.629)			.001			–9.500 (–15.312 to –3.688)				.001		
		Sensory	–2.118 (–3.455 to –.781)			.002			–2.125 (–3.464 to –.786)				.002		
		Relating	–1.701 (–4.507 to 1.106)			.24			–1.625 (–4.444 to 1.194)				.26		
		Body and object use	–2.724 (–5.475 to .026)			.052			–2.750 (–5.495 to –.005)				.05		
		Language	–.627 (–1.768 to .513)			.28			–.625 (–1.771 to .521)				.29		
		Social and self-help	–2.184 (–5.474 to 1.106)			.19			–2.375 (–5.652 to .902)				.16		
	Childhood Autism Rating Scale			–2.389 (–4.550 to –.227)			.03			–2.375 (–4.544 to –.206)				.03	
	**Go/no-go task**														
		Accuracy (%)	6.737 (2.150 to 11.323)			.004			6.900 (2.300 to 11.500)				.003		
		Reaction time (s)	–5.974 (–18.903 to 6.955)			.37			–5.437 (–18.350 to 7.475)				.41		

^a^Coefficient of generalized estimating equation model.

#### Intervention Effects on Behavioral Performance by Go/No-Go Task Analysis

The go/no-go task analysis was used to evaluate the response inhibition ability of children. The GEE analysis showed no significant interaction between time and group effects on accuracy and reaction time (β=2.258; *P=*.11; β*=*–3.105; *P=*.32). However, significant difference in improvements of accuracy could be observed between the 2 groups (β=2.923; *P=*.04). No significant improvement was observed for reaction time (β=–3.171; *P=*.31). However, when stratifying by sex, only girls showed a significant improvement of accuracy (Table 2).

#### Intervention Effects on ADHD Symptoms of Children With Comorbid ADHD

The ADHD-RS-IV scores of 53 children with comorbid ADHD were included in the analysis. Except for the inattention subscale, GEE revealed a significant group × time interaction for total and hyperactivity-impulsivity (β=–2.039 and –1.131; *P=*.03 and .04 respectively). Similarly, compared with the control group, the intervention group showed a significant increase in total and hyperactivity-impulsivity for ADHD-RS-IV scores after the intervention (β=–2.039 and –1.269; *P=*.03 and .02, respectively). This finding was only statistically significant for boys in sex-stratified analysis (see Table 3).

**Table 3:** Effects of the intervention on Attention-Deficit/Hyperactivity Disorder Rating Scale-IV analyzed by generalized estimating equations.

Attention-Deficit/Hyperactivity Disorder Rating Scale-IV outcomes		Group by time interaction effect				Change in baseline intervention group versus control group		
		β^a^ (95% CI)		*P* value		β (95% CI)		*P* value
**Total sample population^b^**								
	Total	–2.039 (–3.928 to –.151)	.03		–2.039 (–3.928 to –.151)		.03	
	Inattention	–.868 (–2.148 to .412)	.18		–.770 (–2.048 to .508)		.24	
	Hyperactivity-impulsivity	–1.131 (–2.189 to –.074)	.04		–1.269 (–2.341 to –.197)		.02	
**Boys^c^**								
	Total	–2.222 (–4.344 to –.101)	.04		–2.222 (–4.344 to –.101)		.04	
	Inattention	–.990 (–2.467 to .488)	.19		–.889 (–2.366 to .589)		.24	
	Hyperactivity-impulsivity	–1.185 (–2.357 to –.014)	.047		–1.333 (–2.519 to –.148)		.03	
**Girls^d^**								
	Total	–.200 (–2.628 to 2.228)	.87		–.200 (–2.628 to 2.228)		.87	
	Inattention	.400 (–.912 to 1.712)	.55		.400 (–.912 to 1.712)		.55	
	Hyperactivity-impulsivity	–.600 (–2.386 to 1.186)	.51		–.600 (–2.386 to 1.186)		.51	

^a^Coefficient of generalized estimating equation model.

^b^53 children with comorbid attention-deficit/hyperactivity disorder were included in the analysis.

^c^45 boys with comorbid attention-deficit/hyperactivity disorder were included in the analysis.

^d^8 girls with comorbid attention-deficit/hyperactivity disorder were included in the analysis.

## Discussion

### Principal Findings

In this randomized controlled trial, we compared the effects of VR-CBT plus LSP intervention with LSP-only treatment in preschool children with ASD. The dropout rate in the intervention group in our study was lower than that reported in previous wearable digital intervention studies (18% vs 33%, respectively) [36], thereby highlighting that nonwearable digital therapies are acceptable for preschool children with ASD. One of the key findings from this work was that compared with the children in the control group, those in the intervention group exhibited greater changes in sensory, relating, body and object use, and self-care areas of the ABC scale. The second encouraging result was the significant differences in the change of total CARS scores between the 2 groups. These results reflect that VR-CBT can improve symptoms of ASD in social skills, repetitive stereotyped behaviors, sensory process disorder, and motor stimulation.

Social skill impairment is one of the core symptoms of children with ASD. As a result, these children also have obvious obstacles in establishing intimate relationships with others and maintaining eye contact [1]. Our results show that VR-CBT simulates human interaction in children with ASD in the real world. Children in the intervention group obtained bidirectional feedback through the virtual platform and therapists. Thus, they gradually had the ability to express themselves and cope with social interactions in real life. Correspondingly, cognitive behavioral training of VR technology is an integration of cognitive and behavioral approaches that replace negative or ineffective thinking and behavioral patterns with structured strategies, which effectively improve mood and adaptive function, helping children with ASD make specific thoughts, feelings, or targeted behavioral changes [37]. In our study, after children mastered the same game, the therapists switched scenes. Children received new trainings and were given different training opportunities that required the utilization of skills mastered in the previous settings, which easily allowed for consistency across methods and techniques throughout the study. This enhanced participants’ interest and reduced their repetitive stereotyped behaviors, thus effectively improving their core symptoms. However, there were no differences in the language scores in the ABC scale between the intervention and the control groups. A potential reason for this could be that only specific languages were used in the VR-CBT intervention for instructions on how to play the game.

Most ASD-related intervention studies typically use parent-rated or clinician-rated symptom measures (ie, psychological scales) to evaluate the effect of the intervention, which is subjective. Given that a go/no-go task is a feasible psychological experiment to identify the behavior of children with ASD [38,39], our research demonstrates that the go/no-go task incorporated in this trial can increase the measurement accuracy of each child. These findings add to the evidence that VR-CBT has a positive effect on response inhibition for pediatric patients with ASD.

The VR-CBT intervention also works more efficiently than single treatment regimens in children with both ASD and ADHD. ADHD is the most common comorbidity in children with ASD, occurring in 25%-81% of this patient population [40,41]. In our trial, 53 (68%) children with ASD also had ADHD. However, studies have shown that these children are currently less likely to be identified and receive appropriate treatment than children with only ADHD [42]. Considerable knowledge gaps still exist in diagnosing and treating children with ASD and ADHD. A study pointed out that executive function deficits in children with ASD are related to social and cognitive deficits, which seriously impair the children’s social adaptation ability [43]. Executive function deficits are also commonly experienced in children with ADHD. CBT embedded in VR provides children with structured and individualized activities that reinforce their ability to maintain attention, thereby exploiting their strengths while addressing their weaknesses [44]. In our trial, children’s impulsivity and response inhibition were reduced through the interaction of VR scenes, which not only improved the symptoms of ADHD but also promoted children’s cognitive flexibility and response inhibition ability. Future studies should focus on constructing a VR comprehensive intervention system from the aspects of working memory and task initiation so as to meet the needs of more pediatric patients.

We noted that the results of the girls differed from those of the boys. A strong male bias in ASD prevalence has been observed in many studies [45]. Related interpretations suggest that males with ASD are found to exhibit more externalizing behavior issues than female patients, such as aggressive behavior, hyperactivity, reduced prosocial behavior, and increased repetitive/restricted behaviors and interests [46,47]. High-functioning females with ASD tend to develop compensation strategies such as camouflage [48]. The obvious improvement of hyperactivity-impulsivity symptoms in boys with comorbid ADHD in this study reflects the gender-specific susceptibility. Osório et al [49] demonstrate female-specific profiles in sensory processing difficulties in ASD. The sensory score changes in female participants might provide a new clue, although the small sample of girls with ASD compromises the statistical power of the analyses.

A previous study has illustrated the critical importance of early detection and treatment for the prognosis of ASD [50]. However, educating children with autism in China is fraught with difficulties, including a dearth of public schools, disorganized private schools, haphazard curriculum designs, and a shortage of teachers with advanced degrees [51]. Those who teach children with ASD also face a lot of challenges [52]. Additionally, caregivers of children with ASD often experience a great deal of stress as well as heavy parenting burdens. The results of a web-based survey of parents of children with ASD indicated that the needs of Chinese families for adjuvant therapy technology are similar to those of families in other countries. However, they have a more diverse spectrum of worries about applying new therapy options such as the ability to pay, privacy breaches, and possible side effects, which lead to low awareness and poor utilization of novel digital therapies [53]. There is a need to consider the practicality of the increasing quantity and types of digital technology for ASD in order to avoid accidentally expanding the digital divide in the worldwide population with ASD [54,55]. Standard procedures should be developed to facilitate the successful maintenance of individuals with ASD. Goncalves et al [56] showed that VR based on surround-screen projection systems are low-cost devices and can be a feasible alternative to head-mounted displays. In this study, cognitive behavioral training based on VR technology is a new type of digital therapy, which is convenient and economical due to its promotion of rehabilitation in early autism. According to the World Health Organization, screen time of preschool children should be no more than 1 hour [57]. Instead of continuously staring somewhere, children were required to participate in a 20-minute VR-CBT session that involved thinking, jogging, and interacting with therapists. During the study period, none of the children reported any adverse event, including dizziness, fatigue, and nausea. Therefore, we believe this intervention can reduce the economic burden and safety concerns of families of children with ASD.

### Limitations

Our study does have some limitations and unexpected findings that should be addressed in future research. First, this trial was performed in a single hospital of Shanghai, which might cause selection bias and fail to be fully representative of the general preschoolers with autism. Second, VR-CBT was based on cartoon games, intended to enhance the ability of children with ASD in order to adapt to the external environment and obey instructions. After the study, some parents and children suggested that scenes with more varieties could improve the effectiveness of the therapy. Our research team agrees with this suggestion, and the second phase of DHI will focus on everyday life skills such as dressing and washing as well as social and communication skills. What’s more, considering the compliance and cooperation of children with ASD, we mainly recruited children with IQ>70 in this study. However, nearly half of the individuals with ASD have lower IQ [58]. Therefore, a digital therapy intervention suitable for a wide range of patients with ASD is an issue worth considering in future research. We believe these factors would be examined in future larger studies by improving equipment and by further assessing the long-term impact of the intervention.

### Conclusions

Our study represents the first randomized controlled trial of a nonwearable digital therapeutic intervention in preschoolers with ASD in China. Our data provide preliminary evidence that the intervention we have implemented using VR-CBT may confer a number of benefits such as significant changes in core symptoms and functions of sensory, motor, and response inhibition of preschoolers with ASD. VR-CBT also showed improvements in the symptoms, including impulsivity and hyperactivity in preschoolers with comorbid ADHD, compared to those in the control group. Although the positive significance of these findings remains to be confirmed in future studies, results from this trial underscore the potential of nonwearable digital therapy as an effective adjunctive therapy in preschoolers with ASD.

## Appendix

Multimedia Appendix 1 Process of the virtual reality–incorporated cognitive behavioral therapy intervention.

Multimedia Appendix 2 Measurement of end point indicators at different time points.

Multimedia Appendix 3 CONSORT-EHEALTH (Consolidated Standards of Reporting Trials of Electronic and Mobile eHealth Applications and Online Telehealth; version 1.6.1).

Multimedia Appendix 4 Mean (SE) score change from the scores before the intervention to those after the intervention in the Autism Behavior Checklist of each group in the intention-to-treat population.

## Abbreviations

ABC: Autism Behavior Checklist
ADHD: attention-deficit/hyperactivity disorder
ADHD-RS-IV: Attention-Deficit/Hyperactivity Disorder Rating Scale-IV
ASD: autism spectrum disorder
CARS: Childhood Autism Rating Scale
CONSORT: Consolidated Standards for Reporting Trials
DHI: digital health intervention
GEE: generalized estimating equation
LSP: learning style profile
SCERTS: Social Communication Emotional Regulation Transactional Support
VR: virtual reality
VR-CBT: virtual reality–incorporated cognitive behavioral therapy

## Data Availability

The data sets generated and analyzed during this study are available from the corresponding author upon reasonable request.
