# Supplementary material for: Effects of a Nonwearable Digital Therapeutic Intervention on Preschoolers With Autism Spectrum Disorder in China: Open-Label Randomized Controlled Trial
Source: J Med Internet Res. 2023 Aug 24;25:e45836. doi: 10.2196/45836 (PMC10485722; doi:10.2196/45836)
Supplement: Multimedia Appendix 1 [file jmir_v25i1e45836_app1.pdf]

Process of the virtual reality–incorporated cognitive behavioral therapy intervention

Steps	Photo	Content	Specific actions	Examples of the game
1	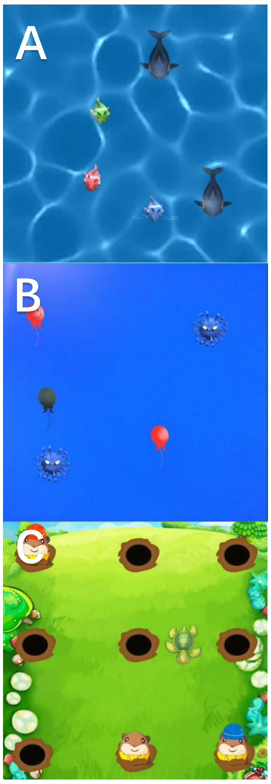	Game perception	VR technology was used to render three scenes of the underwater world, sky balloons, and rural gophers on the colored LED floor tile screen. Participant walked into a virtual scene directly. Then the therapist guided the participant to experience the game and stimulate their initiative to participate in the game. Participant learned to actively respond to external stimuli.	(A). Underwater World: Participant had to step on the red fish without glasses in order to score points. (B). Sky Balloons: Participant was instructed to step on the red balloon with no expression. (C). Rural Gophers: Gophers without glasses or hats were supposed to stepped on. Scores were deducted once the participant made the wrong decision.
2	-	Rule communication	The therapist explained the rules of the game to the participant through short and clear language instructions, encouraging him to be full of curiosity and maintain a sense of participation. After understanding rules and receiving the instruction, participant made a judgment by moving their left and right feet in the visual scene created by VR.	

Steps	Photo	Content	Specific actions	Examples of the game
3	-	Interactive communication	This includes feedback from both the game and the therapist. When the participant's judgments were correct, the virtual scene would intensify their efforts and the therapist would respond based on the performance. The therapist also paid attention to the participant's multiple reactions, created opportunities for dialogue, and allowed the participant to achieve their goals in a gradual manner.	
4	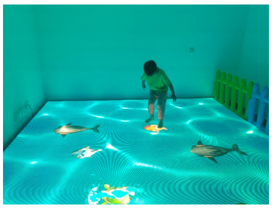 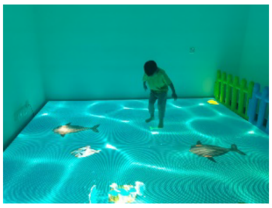	Cognitive training	After the participant received repeated training in the same game, the therapist changed the game scenes to mobilize their attention and cognitive sensitivity. As participant maintained motivation for training, he was able to apply the methods he had previously learned, which helped to improve the participant's self-confidence and promoted their behavioral development.	The participant ran towards the red fish and stepped on it after observing carefully and waiting patiently. Correct actions were rewarded with scores shown in green and if the participant did not choose correctly, points were deducted in red from the score.
5	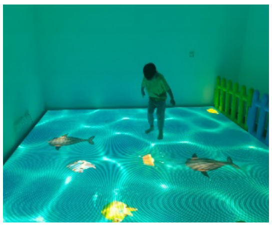	Reaction inhibition	The therapist's instructions transformed the thinking process of interactive communication into specific, visual, and easy-to-understand prompts in the game. This helped the participant to effectively receive and respond to various social information, reducing their impulsive responses and missed responses and improved the accuracy and efficiency of responses. The	When the participant reached 5 points, he received a gold bar (upper right).

Steps	Photo	Content	Specific actions	Examples of the game
			concept of body space could strengthen the participant's understanding of their own bodies and maintain physical control.	
6	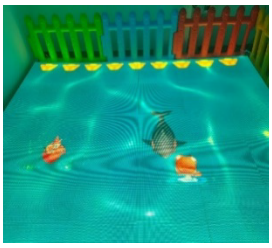	Comprehensive feedback	After participant completed the VR training, he could get feedback from VR-CBT. The therapist might stop or delay the game so as to guide the participant's execution, helping him to make the corresponding correct actions at the right time, and flexibly adapt to the ever-changing external environment.	When the participant played the game according to the instructions, the top corner was filled with gold bars.
