# Supplementary material for: Effects of a Nonwearable Digital Therapeutic Intervention on Preschoolers With Autism Spectrum Disorder in China: Open-Label Randomized Controlled Trial
Source: J Med Internet Res. 2023 Aug 24;25:e45836. doi: 10.2196/45836 (PMC10485722; doi:10.2196/45836)
Supplement: Multimedia Appendix 2 [file jmir_v25i1e45836_app2.pdf]

Measurement of endpoint indicators at different time points

Data	Measure	Informant	T1	T2
Basic information	Age, gender, father and mother's education and etc.	Parents	√	
Primary outcomes	Autism Behavior Checklist (ABC)	Parents	√	√
Secondary outcomes	Childhood Autism Rating Scale (CARS)	Parents	√	√
	ADHD Rating Scale-IV (ADHD-RS-IV)	Parents	√	√
	go/no-go task	Children	√	√

T1: baseline

T2: no more than two weeks after the intervention
