# Supplementary figures and images for: Effects of a Nonwearable Digital Therapeutic Intervention on Preschoolers With Autism Spectrum Disorder in China: Open-Label Randomized Controlled Trial
Source: J Med Internet Res. 2023 Aug 24;25:e45836. doi: 10.2196/45836 (PMC10485722; doi:10.2196/45836)

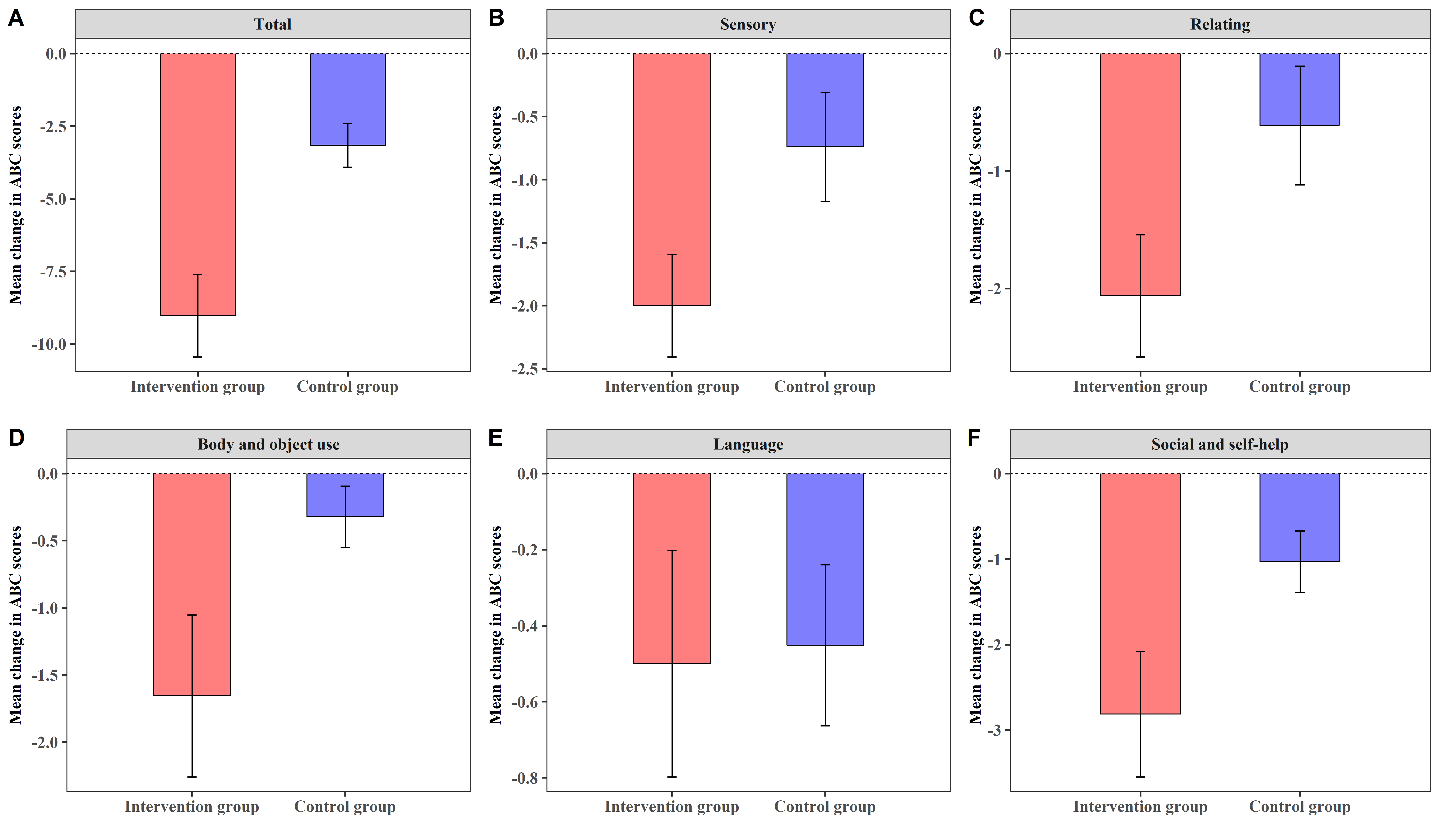

Supplement: Multimedia Appendix 4 [file jmir_v25i1e45836_app4.png]
